# Identification of a Gene Signature Closely Related to Immunosuppressive Tumour Microenvironment Predicting Prognosis of Patients in EGFR Mutant Lung Adenocarcinoma

**DOI:** 10.3389/fonc.2021.732841

**Published:** 2021-09-24

**Authors:** Jia Li, Huahua Li, Chenyue Zhang, Chenxing Zhang, Lifeng Jiang, Haiyong Wang, Huaimin Liu

**Affiliations:** ^1^ Department of Integrated Chinese and Western Medicine, Affiliated Cancer Hospital of Zhengzhou University and Henan Cancer Hospital, Zhengzhou, China; ^2^ Department of Integrated Therapy, Fudan University Shanghai Cancer Center, Shanghai Medical College, Shanghai, China; ^3^ Department of Nephrology, Shanghai Children’s Medical Center, Shanghai Jiao Tong University School of Medicine, Shanghai, China; ^4^ Department of Internal Medicine-Oncology, Shandong Cancer Hospital and Institute, Shandong First Medical University and Shandong Academy of Medical Sciences, Jinan, China

**Keywords:** lung adenocarcinoma, mutation, gene signature, tumour microenvironment, tumour mutation burden

## Abstract

Lung adenocarcinomas (LUADs) harbouring epidermal growth factor receptor (EGFR) mutations are generally unable to benefit from immune checkpoint inhibitors (ICIs) due to an immunosuppressive tumour microenvironment (TME) and a lower tumour mutation burden. Currently, no gene signature can comprehensively evaluate the TME and predict the prognosis of patients with EGFR-mutant LUAD. Using the Cancer Genome Atlas database of EGFR-mutant LUAD based on the immune score derived from the ESTIMATE algorithm, we divided 80 patients with EGFR-mutant LUAD samples into high and low immune score groups with different immune microenvironments. Subsequently, we screened 396 differentially expressed immune-related genes with prognostic value. The top Gene Ontology terms were significantly enriched in biological functions related to T cell differentiation, immune response, cell cycle, and cell proliferation, which are closely related to the immune microenvironment of tumours. In addition, the KEGG pathway enrichment analysis mainly focused on cell cycle, cell adhesion molecules, and cytokine-cytokine receptor interaction, which also had a relationship with the immune response. Subsequently, we identified a three-gene signature including BTLA, BUB1B, and CENPE using the LASSO Cox regression model. The three-gene signature could accurately identify patients at risk of EGFR-mutant LUAD in the training and validation sets and high-risk patients from both the sets exhibited significantly shorter overall survival (*p*=0.0053 and *p*=0.035, respectively). CIBERSORT was used to evaluate the abundance of immune cell infiltration in the EGFR-mutant LUAD microenvironment. The immune activity of B cells and macrophages was higher in the low-risk group, while the immune activity of natural killer cells and T cells was higher in the high-risk group. Thus, the three-gene signature closely related to immunosuppressive TME could predict the risk and prognosis in patients with EGFR-mutant LUAD.

## Background

Lung adenocarcinoma (LUAD) is one of the most common pathological types of non-small cell lung cancer (NSCLC), accounting for approximately half of all lung cancer cases (1). Epidermal growth factor receptor (EGFR) mutations are present in approximately 15% of the LUAD cases in Western populations and in approximately 50% of the cases in Asian populations (2, 3). Patients with EGFR-mutant LUAD showed a significant benefit in terms of progression-free survival with reduced side effects following treatment with tyrosine kinase inhibitors (TKIs). Although TKIs have shown favourable clinical efficacy in advanced LUAD patients with sensitising EGFR mutations, these patients eventually develop therapeutic resistance (4–6). Recently, immunotherapy with immune-checkpoint inhibitors (ICIs) has achieved impressive success and anti-programmed cell death-1 (PD-1)/anti-programmed cell death ligand-1 (PD-L1) inhibitors have been approved by the United States Food and Drug Administration for the treatment of advanced NSCLC. However, patients with EGFR-mutant NSCLC rarely derive a significant benefit from ICI therapy (7, 8).

Several studies have confirmed that EGFR mutations in NSCLC are closely related to an immunosuppressive tumour microenvironment (TME) (9–13) and a lower tumour mutation burden (TMB) (10, 14, 15), which are responsible for an inferior response to PD-1 blockade in NSCLC. EGFR mutations can lead to an uninflamed and immunosuppressive TME with immunological tolerance and weak immunogenicity (10, 16). In addition, an immunosuppressive TME may result from the absence of CD8+ T cell infiltrates and a substantial reduction in TMB (9, 10, 16). Emerging evidence demonstrates that EGFR mutations in NSCLC can affect a number of immune-related genes and induce an immunosuppressive TME (10, 17, 18). Hence, it is highly important to explore immune-related prognostic genes to identify at-risk patients and to reveal the status of the immunosuppressive TME.

Immune-infiltrating cells and stromal cells are major cellular components of a TME (19). The composition of infiltrating immune cells in the TME not only plays a critical role in the progression and aggressiveness of cancer but has also been proposed as an essential prognostic factor (20, 21). Assessing the status of immune infiltrating cells in the TME is expected to help in more accurate diagnosis and prognostic evaluation of tumour patients. Currently, a variety of bioinformatics tools are used to predict the distribution of immune cells by analysing specific gene signatures (22–24). In recent years, studies have shown that EGFR mutations may exert an anti-tumour immune response by affecting the TME (9–13). Patients with EGFR-mutant LUAD have a unique TME, which may be different from that observed in patients with wild-type EGFR. However, in patients with EGFR-mutant LUAD, no signature can comprehensively evaluate the TME based on immune-related genes.

In the present study, we used EGFR mutations and mRNA data of LUAD from the Cancer Genome Atlas (TCGA) to screen the differentially expressed immune-related genes with prognostic value and to compare TMB profiles based on different immune score groups. Gene Ontology (GO) and Kyoto Encyclopaedia of Genes and Genomes (KEGG) enrichment analyses were used to analyse the potential functions of immune-related genes. Subsequently, a three-gene signature closely related to immunosuppressive TME was identified and its potential prognostic value was evaluated and validated. Finally, we explored the relationship between the gene signature and immune cell infiltration in the TME of EGFR-mutant LUAD.

## Materials and Methods

### Data Source and Processing

**Figure 1:**
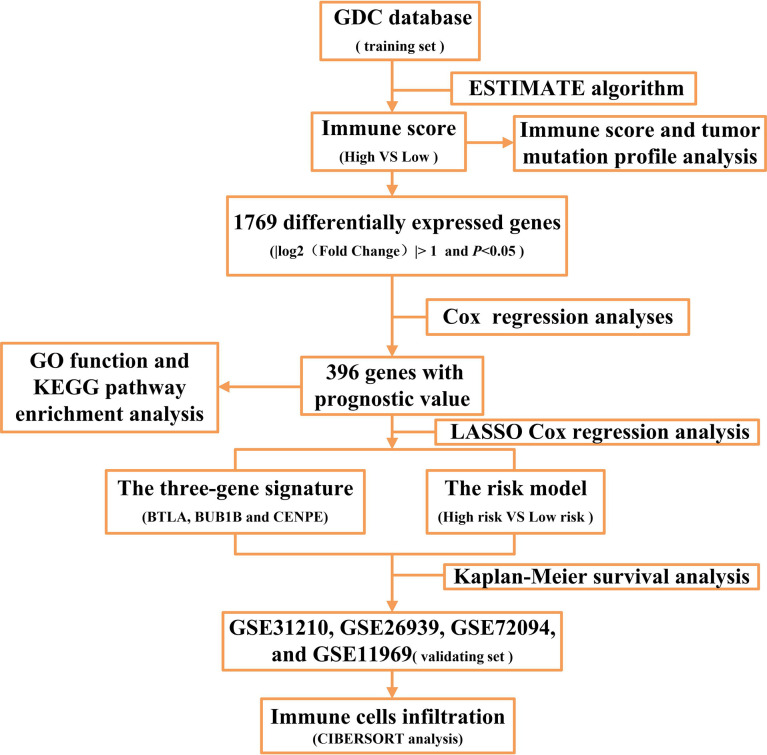
The flow chart of the study.

EGFR mutation and mRNA expression profiling data of 108 patients with LUAD were downloaded from the Genomic Data Commons (GDC) repository (https://portal.gdc.cancer.gov/repository). The TMB data of LUAD patients were obtained from the TCGA pan-cancer study (https://gdc.cancer.gov/about-data/publications/panimmune). Among the 108 patients with EGFR-mutant LUAD, 79 had complete clinical information (**Supplementary Table 1**). Four datasets (GSE31210, GSE26939, GSE72094, and GSE11969) that consisted of 218 patients with EGFR mutations including 212 with clinical information (**Supplementary Table 2**) were downloaded from the Gene Expression Omnibus database (https://www.ncbi.nlm.nih.gov/geo/). A flow chart of this study is shown in **Figure 1**.

### ESTIMATE Algorithm-Derived Immune Scores

The Estimation of Stromal and Immune cells in Malignant Tumours using Expression data (ESTIMATE) algorithm was used to calculate the immune scores based on mRNA expression data. The algorithm was downloaded from the Source Forge software repository (https://sourceforge.net/projects/estimateproject/) (24). A single-sample gene set enrichment analysis was used to generate three scores. Among these, the stromal score indicated the presence of tumour matrix, the immune score indicated tumour immune cell infiltration, and the estimate score indicated tumour purity.

### Screening for Differentially Expressed Immune-Related Genes With Prognostic Value

Among 108 patients with EGFR-positive LUAD, the mRNA expression data of 28 patients could not be downloaded. Therefore, immune scores of 80 patients were calculated according to the mRNA expression data. The immune score for each sample in the training set was calculated according to the ESTIMATE algorithm and the best cut-off value was generated using the X-tile software (25). Using multiples and t-test statistical methods, the differentially expressed genes (1769) were screened between the high and low immune score groups. Data analysis was performed using the LIMMA package (26). The cut-off values for screening differentially expressed genes were |log_2_ (fold change)|> 1 and *p-*value <0.05. Univariate Cox regression was used to determine the prognosis of differentially expressed genes and the threshold was set to *p*<0.05. Altogether, 396 genes with significant prognostic valuewfi 2 were eventually obtained from 1769 differentially expressed genes.

### Enrichment Analysis of Immune-Related Genes With Prognostic Value

Database for Annotation, Visualisation, and Integrated Discovery was used to perform the GO functional analysis and the KEGG pathway enrichment analysis (27). The cut-off value was a false discovery rate of <0.05. GO categories were identified through biological processes, molecular functions, and cellular components.

### Identification of Gene Signature and Construction of a Risk Model

The least absolute shrinkage and selection operator (LASSO) is a better high-dimensional regression classifier for selecting key genes affecting patient prognosis (28). After multiple dimensionality reduction of prognostic genes using the LASSO regression analysis, multiple genomes containing the optimal solution were obtained. The LASSO regression analysis was performed using the publicly available R package.

The optimal prognostic model was constructed, and a formula of risk score was used to evaluate the high-risk and low-risk groups. We obtained the score using the formula Σ_i_ω_i_χ_i_, where ω_i_ and χ_i_ are the coefficient and expression value of each gene, respectively. The risk score for each sample was calculated according to this formula and patients were divided into two groups based on the median risk score. In other words, the score was higher than the median risk score in the high-risk group, while it was lower than the median risk score in the low-risk group.

### Validation of the Validity and Reliability

The GDC dataset from TCGA was used as a training set to analyse the prognostic value of the risk model. Four external datasets (GSE31210, GSE26939, GSE72094, and GSE11969) were used to verify the reliability of the risk model in terms of the prognostic value. Univariate survival analysis of the risk model was performed using the R language (*p*<0.05) (29). Subsequently, we used a survival receiver operating characteristic (ROC) curve to complete the area under the curve (AUC) of the risk model (30).

### Estimation of the Abundance of Immune Cell Infiltration

We quantified the relative abundance of infiltrating immune cells within a complex gene expression mixture using the CIBERSORT platform (https://cibersort.stanford.edu/) (31). The abundance and composition of infiltrating immune cells in a sample were obtained from the gene expression data using CIBERSORT’s deconvolution method.

### Statistical Analysis

Using the survival package of R language, survival analysis was used to compare the survival curves of different risk groups. Cox proportional hazard regression analyses were performed for the prognostic analysis. In addition, the t-test and Wilcoxon test were used to estimate the statistical significance of different groups. Statistical significance was set at *p*<0.05.

## Results

### Screening of Differentially Expressed Immune-Related Genes With Prognostic Value

**Figure 2:**
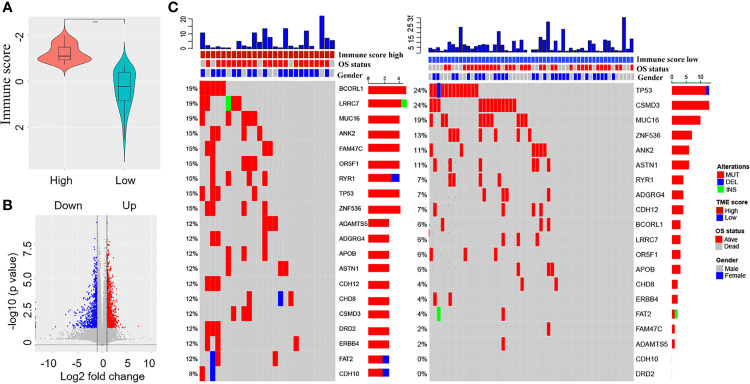
Screening of differentially expressed immune-related genes and comparison of tumour mutation burden (TMB) between the high and low immune score groups. **(A)** Comparison of the immune scores between the high and low immune score groups. **(B)** Screening of differentially expressed immune-related genes between the high and low immune score groups (red colour represents upregulated genes and blue colour represents downregulated genes). **(C)** Comparison of overall survival status, gender, and TMB between the high and low immune score groups.

The immune score could be calculated using immunocyte-related genes in 80 patients with EGFR-mutant LUAD. The Wilcoxon test revealed a significant difference in the immune score between the high immune score group (n=26) and the low immune score group (n=54) (**Figure 2A**, *p*<0.001). Using multiples, t-test statistical methods, and the mRNA expression profiles of 80 patients, differentially expressed genes (1769) were screened between the high and low immune score groups. Among these, 1050 genes were upregulated, and 719 genes were downregulated (**Figure 2B**). Altogether, 396 genes with significant prognostic value were identified using univariate Cox regression.

### Comparison of TMB Between the High and Low Immune Score Groups

According to the TMB data of 80 patients, we assessed the TMB score of each patient and compared the tumour mutation profiles between the high and low immune score groups. The results showed that the TMB score of the low immune score group was higher than that of the high immune score group, although the difference was not statistically significant (Wilcoxon test, *p*=0.07). Differences were observed in mutant genes such as BCORL1 (24% *vs*. 6%), LRRC7 (19% *vs*. 6%), FAM47C (15% *vs*. 2%), OR5F1 (15% *vs*. 6%), and CSMD3 (12% *vs*. 24%) between the high and low immune score groups (**Figure 2C**).

### Enrichment Analysis of Differentially Expressed Immune-Related Genes With Prognostic Value

**Figure 3:**
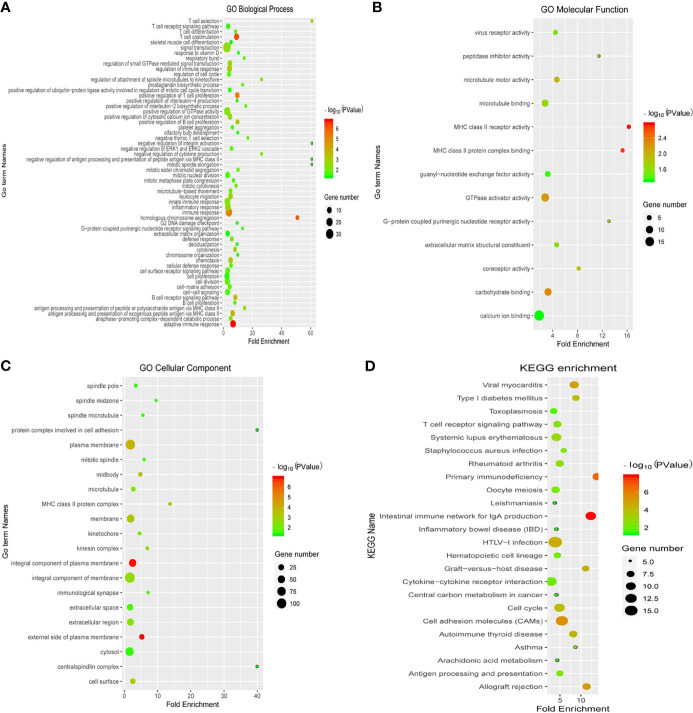
Enrichment analysis of differentially expressed immune-related genes with prognostic value. The Gene Ontology (GO) terminology for biological processes **(A)**, molecular functions **(B)**, and cellular component terms **(C)**, and **(D)** the KEGG pathway enrichment analysis are depicted. Blue colour represents less enrichment to a pathway factor and red colour represents more number of factors enriched in a pathway. Black dots represent the number of enriched factors (the larger the number, the greater the number).

To explore the potential functions of 396 genes with prognostic value, we performed the GO function and KEGG pathway enrichment analyses. The GO terminology for biological processes, molecular functions, and cellular component terms is listed in **Figures 3A–C**. The top GO terms were significantly enriched in biological functions related to T cell differentiation, immune response, cell cycle, and cell proliferation. In addition, the KEGG pathway enrichment analysis was mainly related to cell cycle, cell adhesion molecules, and cytokine-cytokine receptor interactions, which were also related to the immune response (**Figure 3D**).

### Identification of Gene Signature and Construction of a Risk Model

Initially, the LASSO Cox regression model was used to screen the most prognostic genes among the 396 differentially expressed prognostic genes. Thirteen genes (ABCC2, ACAP1, ALG1L, ARHGAP30, ARHGAP9, BHLHA15, BTLA, BUB1B, CDCA6, CCNB2, CD6, CD80, and CENPE) could be used as candidates (*p*=2e−16). Eight of these genes including ABCC2, ARHGAP9, BTLA, BUB1B, CCNB2, CD6, CD80, and CENPE appeared in the subsequent validation set. Therefore, the gene signature and risk models were constructed based on these eight genes. The risk model composed of the aforementioned eight genes could not predict the prognosis of patients. Therefore, all combinations of these eight genes were traversed and gene combinations with significant prognostic value were found. Finally, a three-gene signature (BTLA, BUB1B, and CENPE), which was closely related to immunosuppressive TME, was identified by a random sampling method of 10-cross validation. (Risk score=[−14.66*BTLA]+[1.929*BUB1B]−[2.169*CENPE]). Importantly, we found that the signature constructed by these three genes was the most suitable prognostic model by confirmation and verification. Subsequently, 80 patients were divided into high-risk and low-risk groups according to the median risk score (*Materials and Methods*).

### Evaluating the Prognostic Value of the Gene Signature

**Figure 4:**
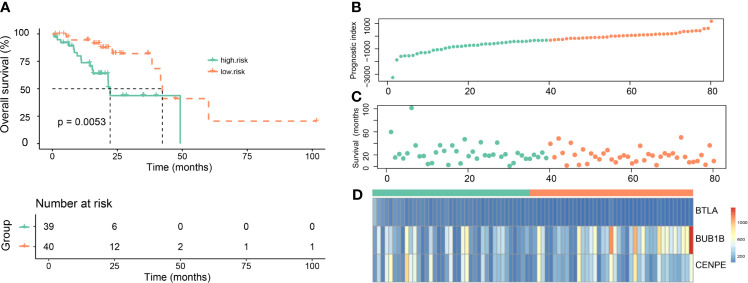
Evaluation of the prognostic value of the gene signature in the Genomic Data Commons dataset from the Cancer Genome Atlas. **(A)** Kaplan–Meier survival curves for overall survival (OS) in the high-risk and low-risk groups. **(B)** The risk score distribution. **(C)** Distribution of patient survival time. **(D)** Heat map of three gene expressions (BTLA, BUB1B, and CENPE).

To further evaluate the prognostic value of the gene signature, Kaplan–Meier survival curves showed that patients in the high-risk group had shorter overall survival than those in the low-risk group (**Figure 4A**, *p*=0.0053). The risk score distribution, number of patients, distribution of patient survival time, and cumulative distribution of survival samples are shown in **Figures 4B, C**. Heat map of the expression of the three genes revealed differences in the expression of these genes between the high-risk group and the low-risk group (**Figure 4D**).

### Validating the Validity and Reliability of the Gene Signature

**Figure 5:**
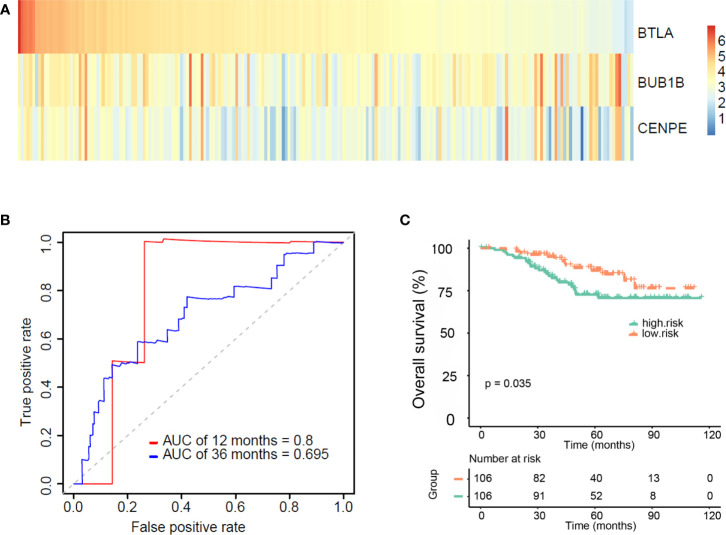
Validating the validity and reliability of the gene signature in four external datasets (GSE31210, GSE26939, GSE72094, and GSE11969). **(A)** Heat map of three gene expressions (BTLA, BUB1B, and CENPE). **(B)** Receiver operating characteristic curves of the gene signature at 12 months and 36 months. **(C)** Kaplan–Meier curves for overall survival (OS) in the high and low-risk groups and number of patients.

Four external datasets (GSE31210, GSE26939, GSE72094, and GSE11969) including 212 EGFR-mutant LUAD patients with clinical information were used as a validation set. Based on the median risk score of the three genes (BTLA, BUB1B, and CENPE) from the training set, 212 patients were divided into the high-risk group and the low-risk group (*Materials and Methods*). **Figure 5A** shows the heat map of the expression of these three genes. The ROC curve was used to assess the prognostic value of the gene signature. The AUCs of the gene signature at 12 months and 36 months were 0.8 and 0.7, respectively (**Figure 5B**). Kaplan–Meier survival curves showed that patients in the high-risk group exhibited shorter OS than those in the low-risk group (**Figure 5C**, *p*=0.035). These results indicate that our gene signature was feasible.

### Estimating the Abundance of Immune Cell Infiltration

**Figure 6:**
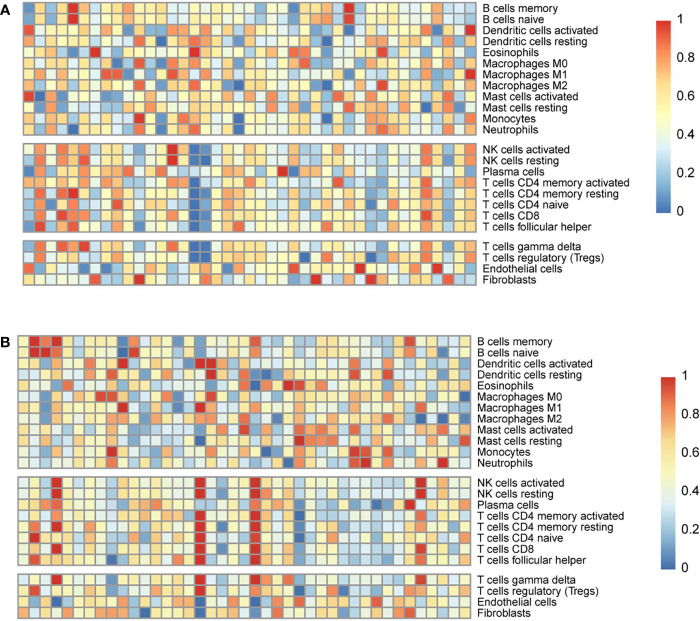
Estimation of the abundance of immune cell infiltration in the Genomic Data Commons dataset from the Cancer Genome Atlas. The abundance of infiltration of 24 types of immune cells in the low-risk **(A)** and high-risk **(B)** groups.

CIBERSORT was used to estimate the abundance of immune cell infiltration. The abundance of infiltration of 24 types of immune cells was normalised as relative proportions in the high-risk and low-risk groups (**Figures 6A, B**). The results showed that the immune activity of B cells and macrophages was higher in the low-risk group, whereas the immune activity of natural killer (NK) cells and T cells was higher in the high-risk group.

In B series cells, which contained memory and naïve B cells. The immune cell score of memory B cells in the low-risk group was higher (0.375 ± 0.004 *vs* 0.351 ± 0.015, *p* =0.121) (mean ± sem). The immune cell score of naïve B cells in the low-risk group was also higher (0.488 ± 0.032 *vs* 0.472 ± 0.054, *p* =0.063) (mean ± sem). Both the immune score of M0 macrophages and M1 macrophages in the low-risk group were higher than those in the high-risk group, and there was a significant difference (0.875 ± 0.036 *vs*. 0.787 ± 0.012, *p*=0.028; 0.522 ± 0.018 *vs*. 0.457 ± 0.004, *p*=0.036) (mean ± sem). In NK series cells, which contained activated and resting cells. Both the immune score of activated *NK* cells and resting NK cells in the high-risk group were higher (0.575 ± 0.035 *vs*. 0.501 ± 0.007, *p*=0.023; 0.564 ± 0.021 *vs.* 0.543 ± 0.004, *p*=0.051) (mean ± sem). Among T series cells, including such as activated CD4 memory T cells, resting CD4 memory T cells, naïve CD4 T cells, CD8 T cells, follicular helper T cells, gamma delta T cells, and regulatory T cells. The immune cell score of the high-risk group were higher those of the high-risk group (0.522 ± 0.018 *vs.* 0.426 ± 0.004, *p*=0.021; 0.486 ± 0.003 *vs.* 0.478 ± 0.005, *p*=0.118; 0.400 ± 0.013 *vs*. 0.384 ± 0.037, *p*=0.070; 0.582 ± 0.026 *vs.* 0.501 ± 0.015, *p*=0.033; 0.513 ± 0.031 *vs.* 0.442 ± 0.005, *p*=0.004; 0.468 ± 0.025 *vs.* 0.231 ± 0.011, *p*<0.001; 0.561 ± 0.030 *vs.* 0.421 ± 0.044, *p*=0.001) (mean ± sem).

## Discussion

EGFR-mutant LUAD is an important molecular subtype that predicts high response rates to TKI therapy (4–6). However, most clinical trials have shown that patients with EGFR mutations cannot benefit from immunotherapy (32–35). Currently, the National Comprehensive Cancer Network guidelines do not recommend immunotherapy for patients with NSCLC harbouring EGFR mutations (7, 8). Several mechanisms responsible for poor response to ICIs have been reported. These include a lower TMB and an uninflamed and immunosuppressive TME (9–16). The PD-1/PD-L1 axis may not be the main immune escape route in EGFR-mutant lung cancer. EGFR activation is possibly responsible for the uninflamed TME in this type of tumour and participates in immunosuppression and immune escape (10, 17, 18). Therefore, a better understanding of the TME and exploring immune-related prognostic biomarkers is expected to help in revealing the molecular mechanism, identifying at-risk groups of patients, and improving clinical outcomes.

Initially, we divided 80 patients with EGFR-mutant samples into the high and low immune score groups with different immune microenvironments. Subsequently, we screened 396 differentially expressed immune-related genes with prognostic value between the high and low immune score groups. The GO and KEGG enrichment analyses were used to analyse the potential functions of 396 immune-related genes with prognostic significance. The top GO terms were significantly enriched in biological functions related to T cell differentiation, immune response, cell cycle, and cell proliferation. In addition, the KEGG pathway enrichment analyses were mainly based on cell cycle, cell adhesion molecules, and cytokine-cytokine receptor interaction. Therefore, we speculated that the screened immune-related genes with prognostic value might reveal the status of the immunosuppressive TME.

TMB plays an important role in predicting response to tumour immunotherapy (36, 37) and immunosuppressive TME results in a substantial reduction in the TMB in NSCLCs (10, 16). We compared the TMB profiles between different immune score groups. The TMB score of the low immune score group was higher than that of the high immune score group (*p*=0.07). Moreover, differences were observed in mutant genes such as BCORL1 (24% *vs*. 6%), LRRC7 (19% *vs*. 6%), FAM47C (15% *vs*. 2%), OR5F1 (15% *vs*. 6%), and CSMD3 (12% *vs*. 24%) between the high and low immune score groups. EGFR-mutant LUAD with different immune score not only has a differentiated TME but also exhibits heterogeneity in the microenvironment. Based on the classification method of immune scores, we observed differences in the TMB profiles between different immune score groups, which also indicates a difference in the immune microenvironment status.

Many studies have demonstrated that EGFR mutations affect immune-related genes to exert a series of biological effects such as immunosuppression and immune escape (38–40). We identified 396 immune-related genes with prognostic value and constructed a three-gene (BTLA, BUB1B, and CENPE) signature. Our three-gene signature could effectively divide EGFR-mutant LUAD patients in the training and validation sets into high-risk and low-risk groups. The high-risk patients exhibited a shorter OS. In addition, ROC curves confirmed the robust prognostic value of the three-gene signature in the training and validation sets. These findings confirmed that our TME-related three-gene signature had a great and reliable prognostic value in patients with EGFR-mutant LUAD.

Infiltrated immune cells are an important part of the TME and play an important role in the formation of the TME (41, 42). Detailed characterisation of immune infiltrating cells in the TME is expected to be more conducive to accurate diagnosis and prognostic evaluation (20, 21). We evaluated the abundance of immune cell infiltration in the EGFR-mutant LUAD microenvironment. The immune activity of B cells and macrophages was higher in the low-risk group, while the immune activity of NK cells and T cells was higher in the high-risk group. These results confirm that the three-gene signature was closely related to the TME and could provide a reference for response to immunotherapy.

All three genes in our signature model have been proven to be related to tumour progression. BTLA is a recently discovered immunosuppressive receptor of the CD28 superfamily in addition to CTLA-4 and PD-L1/PD-1 (43). BTLA is mainly expressed in pulmonary carcinoma cells but shows low expression in tumour-infiltrating lymphocytes. BTLA overexpression is a risk factor for poor prognosis in NSCLC and may be a novel therapeutic target for immunotherapy (44). BUB1B plays a critical role in mitotic checkpoint signalling and chromosome congression, which are closely related to tumourigenesis (45–47). BUB1B overexpression may serve as a predictive marker for LUAD and provide a new potential therapeutic target for inhibiting metastasis of LUAD (48). CENPE is an essential plus end-directed microtubule motor that aligns chromosomes on the metaphase plate (49, 50). CENPE is highly expressed in NSCLC and its high expression is related to poor prognosis (51). Although BUB1B and CENPE have not been reported to be involved in tumour immunity, their functions in the TME warrant further research.

The present study has some limitations. The study involved only bioinformatics-related retrospective research. Hence, prospective clinical sample validation is still needed. The sample size of EGFR-mutant LUAD in our training set was small and some samples lacked complete clinical information. We need to expand the sample size or screen more databases to verify the accuracy and clinical value of the three-gene signature. Due to the lack of data on EGFR-mutant LUAD patients treated with ICIs, we could not determine the relationship between the signature and the response to immunotherapy.

## Conclusions

We identified a three-gene signature with a robust prognostic value based on the immune scores. Importantly, our signature may represent the TME status in patients with EGFR-mutant LUAD. The signature demonstrated a close association with TMB. Our study provides a novel insight into the prognostic stratification of patients with EGFR-mutant LUAD and provides an in-depth understanding of the TME status for immunotherapy in patients with EGFR-mutant LUAD.

## Data Availability Statement

The datasets presented in this study can be found in online repositories. The names of the repository/repositories and accession number(s) can be found in the article/**Supplementary Material**.

## Ethics Statement

The studies involving human participants were reviewed and approved by the Affiliated Cancer Hospital of Zhengzhou University and Henan Cancer Hospital. Written informed consent for participation was not required for this study in accordance with the national legislation and the institutional requirements.

## Author Contributions

HML and HW designed the project and proposed the idea. HHL carried out data download and literature collection. CYZ conducted bioinformatics analysis. CXZ and LJ conducted chart and statistical processing. JL wrote manuscript and processed the data. All authors contributed to the article and approved the submitted version.

## Funding

This study was supported by Joint Fund Project (NSFC-Henan United Fund.U1704181); General Project of Henan Provincial Natural Science Foundation (No.202300410450); Major Special Project of Henan Province Traditional Chinese Medicine Scientific Research Special Project (No.20-21ZYZD07); Key R&D and promotion projects in Henan Province (212102310342).

## Conflict of Interest

The authors declare that the research was conducted in the absence of any commercial or financial relationships that could be construed as a potential conflict of interest.

## Publisher’s Note

All claims expressed in this article are solely those of the authors and do not necessarily represent those of their affiliated organizations, or those of the publisher, the editors and the reviewers. Any product that may be evaluated in this article, or claim that may be made by its manufacturer, is not guaranteed or endorsed by the publisher.

## Supplementary Material

Supplementary Table 1 Clinical information of patients with epidermal growth factor receptor-mutant lung adenocarcinoma from the Genomic Data Commons database.

Supplementary Table 2 Clinical information of patients with epidermal growth factor receptor-mutant lung adenocarcinoma from the Gene Expression Omnibus datasets.

## Abbreviations

LUAD, lung adenocarcinomas; EGFR, epidermal growth factor receptor; ICIs, immune checkpoint inhibitors; TME, tumour microenvironment; TMB, tumour mutation burden; TCGA, the Cancer Genome Atlas; ESTIMATE, Estimation of stromal and immune cells in malignant tumours using expression data; GO, gene ontology; KEGG, Kyoto Encyclopaedia of Genes and Genomes; LASSO, least absolute shrinkage and selection operator; ROC, receiver operating characteristic; NK, natural killer; NSCLC, non-small cell lung cancer; TKIs, tyrosine kinase inhibitors; PD-1, anti-programmed cell death-1; PD-L1, anti-programmed cell death ligand-1; AUC, area under the curve.
