# Supplementary material for: Identification of a Gene Signature Closely Related to Immunosuppressive Tumour Microenvironment Predicting Prognosis of Patients in EGFR Mutant Lung Adenocarcinoma
Source: Front Oncol. 2021 Sep 24;11:732841. doi: 10.3389/fonc.2021.732841 (PMC8498106; doi:10.3389/fonc.2021.732841)
Supplement: Supplementary Table 1 — Clinical information of patients with epidermal growth factor receptor-mutant lung adenocarcinoma from the Genomic Data Commons database. [file Table_1.docx]

Table1.

| Clinical factors | The number of patients (n=79) |
| --- | --- |
| Sex |  |
| Male | 33 |
| Female | 46 |
| AJCC stage |  |
| Stage I | 32 |
| Stage II | 22 |
| Stage III | 18 |
| Stage IV | 6 |
| Survival status |  |
| Alive | 54 |
| Dead | 25 |
| Age (median,year) | 40-86 (70) |
| Overall Survival (median,month) | 0.59-101.64 (18.17) |
