# Supplementary material for: Identification of a Gene Signature Closely Related to Immunosuppressive Tumour Microenvironment Predicting Prognosis of Patients in EGFR Mutant Lung Adenocarcinoma
Source: Front Oncol. 2021 Sep 24;11:732841. doi: 10.3389/fonc.2021.732841 (PMC8498106; doi:10.3389/fonc.2021.732841)
Supplement: Supplementary Table 2 — Clinical information of patients with epidermal growth factor receptor-mutant lung adenocarcinoma from the Gene Expression Omnibus datasets. [file Table_2.docx]

Table2

| Clinical factors | The number of patients (n=212) |
| --- | --- |
| Sex |  |
| Male | 77 |
| Female | 127 |
| Unkown | 8 |
| Survival status |  |
| Alive | 173 |
| Dead | 39 |
| Age (median, year) | 35-85 (62) |
| Overall Survival (median, month) | 0.133-115.53 (53.2) |
